# Ethnobiological notes and volatile profiles of two rare Chinese desert truffles

**DOI:** 10.1080/21501203.2022.2035005

**Published:** 2022-02-14

**Authors:** Bin Lu, Feng-Ming Zhang, Fu-Qiang Yu, Andrea C. Rinaldi

**Affiliations:** a Key Laboratory for Forest Resources Conservation and Use in the Southwest Mountains of China, Southwest Forestry University, Kunming, China; b The Germplasm Bank of Wild Species, Yunnan Key Laboratory for Fungal Diversity and Green Development, Kunming Institute of Botany, Chinese Academy of Sciences, Kunming, China; c Department of Biomedical Sciences, University of Cagliari, Monserrato, Italy

**Keywords:** Wild edible mushrooms, headspace solid-phase microextraction (HS-SPME), aroma, ascomycetes, hypogeous fungi

## Abstract

The production of a distinct profile of volatile organic compounds plays a crucial role in the ecology of hypogeous Ascomycetes, and is also key to their gastronomic relevance. In this study, we explored the aroma components of two rarely investigated Chinese desert truffles, namely *Mattirolomyces terfezioides* and *Choiromyces cerebriformis*, using headspace solid-phase microextraction (HS-SPME) coupled with gas chromatography-mass spectrometry (GC-MS). Our investigation revealed the significant presence of sulphur-containing volatiles in the aroma of *M. terfezioides* but not in *C. cerebriformis*. We discussed available information on the distribution of these interesting truffles in China and their use as choice food by local people.

## 1. Introduction

“Desert truffles” is a cumulative term to indicate a heterogeneous group of “edible hypogeous fungi growing in arid areas throughout the world” (Kagan-Zur et al. 2014). A large number of desert truffles are ascomycetes, linked in mycorrhizal association to a variety of host plants, in reality occurring in a wide range of habitats, including temperate deciduous and coniferous forests. Despite desert truffles are less appreciated from an organoleptic point of view than prized *Tuber* (true truffles), a long historic record exists documenting their use as food in many areas of the world, with a particular emphasis on countries surrounding the Mediterranean basin and in the Middle East (Boa 2004; Martínez-Tomé et al. 2014).

Recently, the presence of two interesting desert truffles has been confirmed in China, using a mix of morphological and molecular approaches. The first one is *Mattirolomyces terfezioides* (Mattir.) E. Fisch. [MB#255072], the type species of *Mattirolomyces* E. Fisch. (*Pezizales, Pezizaceae*). This mushroom has a prevalently European distribution (Gógán Csorbainé et al. 2009; Kovács et al. 2009; Assyov and Slavova 2016), but has been reported also from India, South Korea, and China, where the conspecificity of specimens from the northern part of the country with European samples has been demonstrated (Wang et al. 2017). *M. terfezioides* is described to possess a peculiar sweet flavour and a distinctive taste, and is considered a truffle with significant potential as gastronomic delicacy (Gógán Csorbainé et al. 2009; Assyov and Slavova 2016). The other species of interest is *Choiromyces cerebriformis* (*Pezizales, Tuberaceae*), a new taxon that was described from Yunnan Province, linked to *Pinus yunnanensis* (Yuan et al. 2021). The genus *Choiromyces* hosts at least one well-known edible species, namely *C. meandriformis* Vittad (syn. *C. venosus* (Fr.) Th. Fr.), with a long culinary tradition in Sweden and Central-East Europe (Gógán Csorbainé et al. 2009; Wedén et al. 2009).

Knowledge on desert truffles in China is scant. The recent collection of samples of *M. terfezioides* and *C. cerebriformis* by members of our team offered the opportunity to investigate an aspect that has received very little attention so far, i.e. the identity of aroma components of these edible mushrooms. Indeed, volatile organic compounds (VOCs) play a crucial role in the ecology of hypogeous ascomycetes, and also in determining their appreciation as “gourmet food”, a fact well studied in *Tuber* but virtually ignored for desert truffles (Splivallo et al. 2011; Lu et al. 2021).

## 2. Materials and methods

### 2.1 Fungal materials and identification

**Figure 1.**
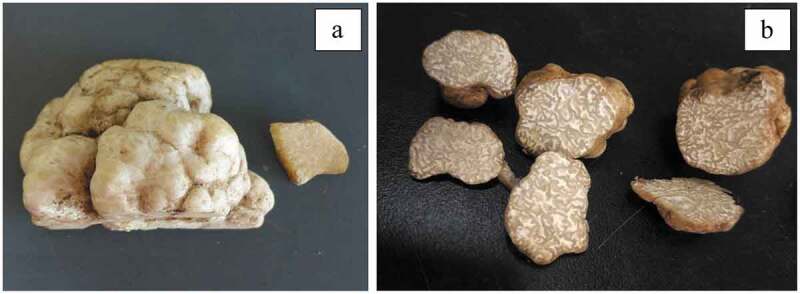
Sporocarps of Chinese desert truffles *Mattirolomyces terfezioides* (a) and *Choiromyces cerebriformis* (b).

Fresh sporocarps of *M. terfezioides* and *C. cerebriformis* were collected from Huaiyang County, Zhoukou City, Hebei Province, and Yongren County, Chuxiong State, Yunnan Provinces of China, respectively. The former was harvested in a cornfield, and the latter distributed in a *Pinus yunnanesis* forest. They were wrapped in tin foil, placed in a ziplock bag and brought back to the laboratory for further analysis. Sporocarps were identified on the basis of published descriptions of macroscopic and microscopic characters as *M. terfezioides* and *C. cerebriformis* (Figure 1) (Wang et al. 2017; Yuan et al. 2021). Molecular characterisation of sporocarps using an approach based on PCR amplification and sequencing of the complete internal transcribed spacer (ITS) regions in nuclear ribosomal DNA (Gardes and Bruns 1993), confirmed the identification. The relevant sequences were deposited in GenBank under the following access codes: *M. terfezioides*, MT890667; *C. cerebriformis*, MT890668. As for *M. terfezioides*, our sequence (MT890667) has 100% similarity with several other sequences attributed to this species, originating both from Asia (KT963177) and Europe (AJ305045, AJ272444). The sequence of *C. cerebriformis* we present as a prove of sporocarp identification, MT890668, has been originally deposited in GenBank as *C. helanshanensis*, but if blasted it results very similar (>98%) to MT672014, MT672013 and MW209701, i.e. the sequences deposited (as *Choiromyces* sp.) by Yuan and colleagues before they described *C. cerebriformis*, and that were used to discriminate this species from *C. helanshanensis* (see Yuan et al. 2021). Total DNA was extracted from fresh fruitbody using the CTAB method. The ITS1F and ITS4 primers were used to amplify the internal transcribed spacers 1 and 2 with the 5.8S rDNA (ITS) (Gardes and Bruns 1993). PCR reactions were conducted on an ABI 2720 Thermal Cycler (Applied Biosystems, Foster City, CA, USA) or an Eppendorf Master Cycler (Eppendorf, Netheler-Hinz, Hamburg, Germany). The PCR programs were as follows: pre-denaturation at 94°C for 5 min, then followed by 35 cycles of denaturation at 94°C for 50s, annealing at 50°C elongation at 72°C for 60s, a final elongation at 72°C for 8 min was included after the cycles. PCR products were purified with a Gel Extraction & PCR Purification Combo Kit (Spin-column) (Bioteke, Beijing, China) and then sequenced on an ABI-3730-XL sequence analyser (Applied Biosystems, USA) using the same primers as in the original PCR amplifications. Use the Basic Local Alignment Search Tool (BLAST) algorithm to analyse sequence similarity through the National Center for Biotechnology Information (NCBI) website http://www.ncbi.nlm.nih.gov/), and retrieve the similarity from GenBank 99% DNA sequence.

### 2.2 Chemicals

A divinylbenzene/carboxen/polydimethylsiloxane (DVB/CAR/PDMS) Stable Flex fibre of 50/30 *μ*m was purchased from Supelco, USA and used for headspace solid-phase microextraction (HS-SPME).

### 2.3 HS-SPME

For each SPME analysis, the vial was closed with a PTFE silicon septum and made air tight. Keep the vial indoors. The fibres were then exposed to the headspace of the sample for 30 min without heating, at room temperature (about 25°C). The fibre was then removed from the vial and placed in the injection port of the gas chromatogram. A desorption time of 10 min with the injection temperature of 250°C was adequate to desorb most of the analytes from the fibre under experimental conditions. After desorption from the fibre, the extract was directly transferred to the analytical column. The fibres were cleaned daily to prevent contamination.

### 2.4 Gas chromatography-mass spectrometry (GC-MS) analysis

Analysis of volatile compounds was carried out using gas chromatography-mass spectrometry (GC-MS, Agilent Technologies, Agilent 7890A along with Agilent 5975C, USA). Chromatographic separation was performed on a DB-5 MS capillary column (30 m × 0.25 mm ID, 0.25 *μ*m film thickness, Agilent Technologies, USA). The following chromatographic program was used: the oven temperature was held for 2 min at 35°C, then increased to 125°C at a rate of 3°C/min (held for 1 min), then a rate of 20°C/min increased to 250°C (held for 1 min). The carrier gas (He) flow was constant at 1 mL/min. The injection port was operated in the splitless mode at 250°C. The operating conditions for the MS system were as follows: the ion source temperature was 230°C, electron ionisation mode at 70 eV. Full scan mode with a scan range of m/z 40–500 was used for data acquisition. Each component was subjected to NIST11 library search and data were analysed by using MSD ChemStation software (Agilent Technologies, version G1701EA E. 02. 02. 1431). For each analyte, its relative mass fraction was calculated by peak area normalisation method.

### 2.5 Evaluation of volatile aroma components

For the measurement of retention indices (RI), a mix of n-alkanes ranging from hexane to triacontane was used (Sigma-Aldrich Co., St. Louis, MO, USA). The identification of volatiles of the truffle samples was subjected to NIST11 library search and the mass spectral match factor (similarity >80) was used to judge whether a peak was correctly identified or not. For the determination of the retention index (RI), a series of n-alkanes (C7 to C40) were used under the same experimental conditions. The computational formula of RI is as follows:

$$RI=100\times \left[n+\frac{RT_{R\left(x\right)}-RT_{R\left(n\right)}}{RT_{R\left(n+1\right)}-RT_{R\left(n\right)}}\right]$$

where n and (n + 1) are respectively the number of carbon atoms in alkanes eluting before and after the compound, RT_R(n)_ and RT_R(n+1)_ are the corresponding retention time and RT_R(x)_ is the retention time of the compound to be identified (RT_R(n)_ < RT_R(x)_ < RT_R(n + 1)_).

Relative odour activity value (ROAV) was used to evaluate the contribution of each volatile substance to the overall flavour of desert truffles (Gu et al. 2012; Li et al. 2015; Liu et al. 2011). The ROAV_stan_ = 100 is defined as the component that contributes the most to the flavour of the sample, and the ROAV of other volatile components, if present, is less than 100. The calculation formula is as follows:

$$ROAV_{x}=100\times \frac{C_{x}}{C_{stan}}\times \frac{T_{stan}}{T_{x}}$$

in which C_x_ and T_x_ represent the relative content of each volatile compound % and odour threshold (*μ*g/kg), respectively, and C_stan_ and T_stan_ represent the relative concentration of the component at which it makes the greatest contribution to the overall flavour and the corresponding odour threshold (*μ*g/kg), respectively. Relevant available thresholds are from literature references. It is generally believed that aromatic compounds with high ROAV are most likely to be the main contributors to the overall aroma.

## 3. Results and discussion

Desert truffles are hypogeous *Ascomycota* typically found in arid and semi-arid areas throughout the world that have evolved in several lineages within the *Pezizaceae* and *Tuberaceae* (https://ascomycete.org/). Although desert truffles do not attain the astounding commercial prices of their “noble” cousins, the real truffles, such as the white truffle (*Tuber magnatum* Picco) and the Périgord truffle (*Tuber melanosporum* Vittad.), some species have a considerable economic significance for rural populations in several parts of the world (Louro et al. 2019). Being rich in proteins and poor in carbohydrates and lipids, desert truffles fruit bodies are a potentially important source of healthy and nutrient-dense food for both animals and humans. A recent study of the genomes of selected desert truffles has revealed many new, intriguing details on the expression of fungal genes related to sexual reproduction and of both fungal and plant genes implicated in the development and functioning of the mycorrhizal symbiosis that characterises their lifestyle (Marqués-Gálvez et al. 2021).

**Figure 2.**
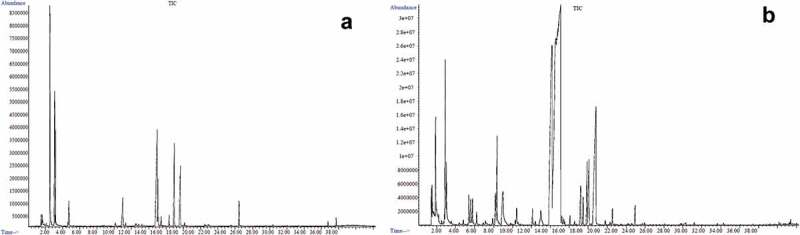
Total ion current (TIC) chromatogram of *Mattirolomyces terfezioides* (a) and *Choiromyces cerebriformis* (b).

**Figure 3.**
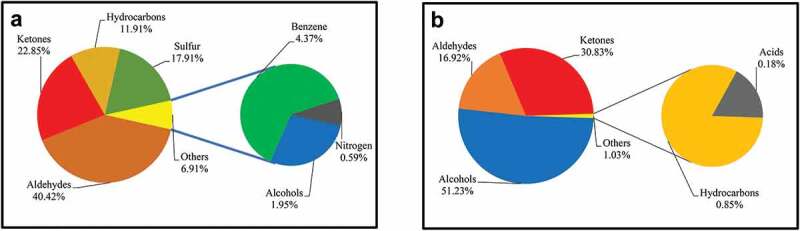
Chemical classes of VOCs and their relative amount from fruit bodies of *Mattirolomyces terfezioides* (a) and *Choiromyces cerebriformis* (b).

According to the comparative search of the mass spectrum of each peak in the obtained chromatogram (Figure 2) and the standard mass spectrum of the NIST 11 library, a total of 31 VOCs was identified in the aroma of the two Chinese desert truffles analysed. Overall, the main components include aldehydes, ketones, olefins, benzene, sulphur compounds, nitrogen compounds, alcohols, and acids, with a markedly distinct profile in each species (Figure 3). As shown in Table 1, VOCs with relatively high content in the aroma of *M. terfezioides* were 3-octanone (22.85%), (methylthio)-ethane (17.00%), phenylacetaldehyde (12.68%), 3-methylbutyraldehyde (11.87%), 2-methylbutyraldehyde (8.28%), D-limonene (7.33%), methylthiopropionaldehyde (5.52%), β-pinene (3.59%), (1-ethylpropyl)-benzene (3.55%), and 2-methyl-1-butanol (1.95%). In the case of *C. cerebriformis*, 1-octene-3-one (29.72%), 1-octen-3-ol (33.65%), and (Z) −2-octene −1-ol (11.74%) were relatively abundant in the relevant aroma profile.

**Table 1.** Compounds detected in Chinese desert truffles by GC-MS (n=3).

No.	Compounds	Molecular formula	RI (DB-5MS)	Relative content/(%)		Threshold (*μ*g/kg)	ROAV	
				*Mattirolomyces terfezioides*	*Choiromyces cerebriformis*		*Mattirolomyces terfezioides*	*Choiromyces cerebriformis*
1	1-Hexen-3-ol	C_6_H_12_O	NC	-	1.45 ± 0.64	NF	-	NF
2	2-Hexen-1-ol, (Z)-	C_6_H_12_O	864	-	4.39 ± 1.30	NF	-	NF
3	1-Octen-3-ol	C_8_H_16_O	987	-	33.65 ± 2.90	1 ^[a, b]^	-	100.00
4	2-Octen-1-ol, (Z)-	C_8_H_16_O	1073	-	11.74 ± 3.96	NF	-	NF
5	1-Butanol, 2-methyl-	C_5_H_12_O	NC	1.95 ± 0.28	-	NF	NF	-
6	Acetaldehyde	C_2_H_4_O	NC	0.38 ± 0.02	1.31 ± 0.35	NF	NF	NF
7	Butanal	C_4_H_8_O	NC	0.26 ± 0.04	-	NF	NF	-
8	Butanal, 3-methyl-	C_5_H_10_O	NC	11.87 ± 0.93	4.80 ± 1.15	9 ^[b]^	7.50	1.58
9	Butanal, 2-methyl-	C_5_H_10_O	NC	8.28 ± 1.76	2.17 ± 0.67	12.5 ^[b]^	3.77	0.52
10	Hexanal	C_6_H_12_O	NC	-	0.61 ± 0.21	9 ^[b]^	-	0.20
11	2-Hexenal, (E)-	C_6_H_10_O	844	-	1.55 ± 0.07	NF	-	NF
12	Heptanal	C_7_H_14_O	NC	0.47 ± 0.11	-	NF	NF	-
13	Methional	C_4_H_8_OS	911	5.52 ± 0.24	0.63 ± 0.25	NF	NF	NF
14	Benzaldehyde	C_7_H_6_O	954	-	1.18 ± 0.56	320 ^[b]^	-	0.01
15	Benzeneacetaldehyde	C_8_H_8_O	1050	12.68 ± 3.40	1.79 ± 0.74	0.7 ^[b]^	103.06	7.60
16	5-Methyl-2-phenyl-2-hexenal	C_13_H_16_O	1493	0.96 ± 0.85	-	NF	NF	-
17	2-Octenal, (E)-	C_8_H_14_O	1058	-	2.40 ± 1.56	3 ^[a, b]^	-	2.38
18	2-Nonenal, (E)-	C_9_H_16_O	1158	-	0.48 ± 0.28	0.4 ^[b]^	-	3.57
19	1-Penten-3-one, 4-methyl-	C_6_H_10_O	NC	-	1.11 ± 0.12	NF	-	NF
20	1-Octen-3-one	C_8_H_14_O	979	-	29.72 ± 5.85	0.8 ^[b]^	-	110.40
21	3-Octanone	C_8_H_16_O	994	22.85 ± 2.56	-	1.3 ^[b]^	100.00	-
22	1-Hexene, 3-chloro-	C_6_H_11_Cl	NC	-	0.85 ± 0.13	NF	-	NF
23	β-Pinene	C_10_H_16_	996	3.59 ± 1.07	-	NF	NF	-
24	3-Octene, (Z)-	C_8_H_16_	1005	0.99 ± 0.22	-	NF	NF	-
25	D-Limonene	C_10_H_16_	1034	7.33 ± 2.15	-	5.9 ^[b]^	7.07	-
26	Benzene, 1-methoxy-3-methyl-	C_8_H_10_O	1022	0.82 ± 0.22	-	NF	NF	-
27	Benzene, (1-ethylpropyl)-	C_11_H_16_	1187	3.55 ± 1.84	-	NF	NF	-
28	Methanethiol	CH_4_S	NC	0.91 ± 0.40	-	4 ^[b]^	1.29	-
29	Ethane, (methylthio)-	C_3_H_8_S	NC	17.00 ± 3.07	-	NF	NF	-
30	Dimethylamine	C_2_H_7_N	NC	0.59 ± 0.08	-	NF	NF	-
31	n-Hexadecanoic acid	C_16_H_32_O_2_	1953	-	0.18 ± 0.10	NF	-	NF

“-”, not detected; “NC”, since the experimental data is not within the retention time range of n-alkanes (C_7_-C_40_), its retention index value cannot be calculated; “NF”, no analysis was made because the odor threshold of the compound could not be found in the literature; a, Li et al., 2015; b, Feng et al., 2019.

In general, only a small proportion of volatile compounds contribute significantly to the overall flavour of any truffles, and some other ingredients may only assist the overall aroma. The contribution of volatile compounds to the aroma of desert truffles, and of any other mushrooms, is determined by their content and odour threshold. Some compounds with low content but low odour threshold may also play an important role in the overall flavour of truffles. The ROAV values of the aroma components of desert truffles according to this test are shown in Table 1. In this study, it was found that there are six main volatiles aroma components (ROAV ≥ 1) of *M. terfezioides*, namely benzeneacetaldehyde, 3-octanone, 3-methyl-butanal, D-limonene, 2-methyl-butanal, methanethiol, that together form the unique smell of *M. terfezioides*. On the other side, the main volatile aroma components of *C. cerebriformis* (ROAV ≥ 1) are 1-octen-3-one, 1-octen-3-ol, benzeneacetaldehyde, (E)-2-nonenal, (E)-2-octenal, 3-methyl-butanal, while hexanal and 2-methyl-butanal play an important role in overall aroma modification (0.1 ≤ ROAV < 1).

As mentioned above, the production of pungent aromas through a combination of VOCs by hypogeous ascomycetes is both of relevance for their ecology – favouring dispersion by animals – and plays a key role in the universal praise of these mushrooms as food delicacies. While the aroma of *Tuber* has been studied in some detail, with a number of studies delving into the volatile profile of a significant number of species (e.g. Vita et al. 2015; Liu and Li 2017; Strojnik et al. 2020), very little is known about the VOCs produced by the sporocarps of desert truffles. A possible, certainly partial, explanation of this apparent lack of interest in the aroma characterisation of desert truffles, is that the flavour of these mushrooms is in most cases much weaker than that of true truffles (*Tuber*), and therefore it is (erroneously) believed that it contributes less to their organoleptic properties. A few studies have focused on the volatiles produced by *Picoa lefebvrei* (Pat.), *Tirmania nivea* (Desf.) Trappe, and *Terfezia boudieri* (Chatain) (Omer et al. 1994; Kamle et al. 2017). The volatile profile of *T. boudieri* and *T. nivea* was characterised by the presence of 1-octen-3-ol and hexanal as main components, but also included branched-chain amino acid derivatives such as 2-methylbutanal and 3-methylbutanal, phenylalanine derivatives such as benzaldehyde and benzenacetaldehyde, and methionine derivatives such as methional and dimethyl disulphide; on the other side, the least aromatic desert truffle, *P. lefebvrei*, contained low levels of 1-octen-3-ol as the main volatile (Kamle et al. 2017).

In a recent study, Claude Murat and colleagues compared the genomes of true truffles (*Tuber magnatum* Pico, *T. melanosporum* Vittad., *T. aestivum* Vittad.) with that of desert truffles *T. boudieri* and *C. venosus* (see above for synonymy). The comparative analysis of genes coding for VOC synthesis revealed that desert truffles showed a low expression of almost all of the sulphur-related volatile organic compounds genes relative to *Tuber*, concluding that the distinct flavour of selected desert truffles may originate from a different set of pathways (Murat et al. 2018). As for *Tuber*, “volatile organic compounds from pungent truffle odours are not the products of *Tuber*-specific gene innovations, but rely on the differential expression of an existing gene repertoire,” noted the authors, adding that “ … specific VOC compositions of individual truffle species may thus be largely explained by the differential expression of selected subsets of metabolic genes, while variation in gene content and/or gene copy number appear to play a relatively minor role in *Tuber* aroma formation,” (Murat et al. 2018).

In an attempt to contribute to the knowledge of aroma constituents in desert truffles, in China and beyond, this study used an unheated experimental design to simulate the temperature in the natural environment, combined with headspace solid-phase microextraction to adsorb and extract *M. terfezioides* and *C. cerebriformis* aroma components, using a gas chromatography-mass spectrometry combined technology. Analysis and detection of the extracted volatile aroma components, according to the type and relative content of VOCs, showed significant differences between the two mushrooms. In particular, sulphur-containing VOCs, like methional, methanethiol and ethane, (methylthio)-, were well represented in the *M. terfezioides* aroma profile, but were absent or scarcely present in that of *C. cerebriformis*. This finding supports the observations of Murat et al. (2018), about the low expression of the genes for sulphur-containing volatile compounds in *C. venosus*. The odour of *C. venosus* has been reported as “very strong and nauseating at maturity …., or spirituous to aromatic that turns unpleasant after drying (Moreno et al. 2012). On the other hand, the flavour of *Mattirolomyces* is described as “agreeable or somewhat spermatic in young and mature specimens, unpleasant in overmature ascomata,” (Assyov and Slavova 2016). No specific data on the spore dispersion of either *Mattirolomyces* or *Choiromyces* exist, but on the basis of what we knew about the ecology of other hypogeous Ascomycota, it can be speculated that also in the case of these desert truffles the spores are either released passively into the soil from disintegrated asci (or, if the sporocarp emerges, spores can be dispersed with the help of wind and water) or are dispersed through animal mycophagy, mainly by mammals but also birds and arthropods (Trappe and Claridge 2005; Bradai et al. 2014). Further work is certainly needed in order to disclose these important aspects of the natural history of desert truffles, but our present study provides a reference basis for the follow-up research on the aroma components of Chinese *Mattirolomyces* and *Choiromyces*, and has certain guiding significance.

Comparing the volatile profiles of *M. terfezioides* and *C. cerebriformis* with that of the many *Tuber* species that have been studied so far, it is not a straightforward matter. In *Tuber*, more than 200 VOCs have been identified collectively, with some 30–60 volatiles typically making the aromatic profile of a single species (Mustafa et al. 2020). Specific odorants common to many white and black truffle (*Tuber*) species are also important constituents of the aroma of *M. terfezioides* and *C. cerebriformis*. This includes 2-methyl-butanal, 3-methyl-butanal, and 1-octen-3-ol, with a typical fungal flavour (Mustafa et al. 2020; Allen and Bennett 2021). Notwithstanding these similarities, any fungal volatile profile is unique, and the present work on Chinese desert truffles confirms this reality.

The known diversity of hypogeous fungi in China is impressive, with more than 220 species recorded so far, hosted in 25 genera of 17 families (Fan 2018). Besides the ecological role of hypogeous Ascomycetes as obligated ectomycorrhizal symbionts of forest trees, of which they improve the nutrient and water uptake and resistance to diseases, some of these mushrooms are sought by local populations as choice food. As for the desert truffles object of this work, *M. terfezioides* was reported sold in the vegetable market of Beijing in 1940s (Liu and Guo 1984; Wang et al. 2017), and is listed among Chinese edible mushrooms (Dai et al. 2010). On the basis of our observations, *C. cerebriformis* was sold by local people as white truffle (*Tuber*) in Yongren County. Another species of *Choiromyces, C. helanshanensis* Juan Chen and P.G. Liu (Pezizales, Tuberaceae) – a taxon described few years back on the basis of material collected in the Helanshan National Nature Reserve, Inner Mongolia, linked to *Picea crassifolia* (Chen et al. 2016) – was said to be eaten by local people in Helanshan Mountain. No other more consumption information about desert truffles in China is currently available, mainly because of the scarce attention researchers have paid to these hypogeous mushrooms, a gap we aim to bridge.
